# Increased modularity of the resting‐state network in children with nonsyndromic cleft lip and palate after speech rehabilitation

**DOI:** 10.1002/brb3.2094

**Published:** 2021-08-03

**Authors:** Hua Cheng, Bo Rao, Wenjing Zhang, Renji Chen, Yun Peng

**Affiliations:** ^1^ Department of Radiology Beijing Children's Hospital National Center for Children's Health Capital Medical University Beijing China; ^2^ Departments of Radiology Zhongnan Hospital of Wuhan University Wuhan China; ^3^ Department of Oral and Maxillofacial Plastic and Trauma Surgery Center of Cleft Lip and Palate Treatment Beijing Stomatological Hospital Beijing China

**Keywords:** graph theory, modularity, nonsyndromic cleft lip and palate, resting‐state functional MRI, speech therapy

## Abstract

**Introduction:**

Speech therapy is the primary management followed the physical management through surgery for children with nonsyndromic cleft lip and palate (NSCLP). However, the topological pattern of the resting‐state network after rehabilitation remains poorly understood. We aimed to explore the functional topological pattern of children with NSCLP after speech rehabilitation compared with healthy controls.

**Methods:**

We examined 28 children with NSCLP after speech rehabilitation (age = 10.0 ± 2.3 years) and 28 healthy controls for resting‐state functional MRI. We calculated functional connections and the degree strength, betweenness centrality, network clustering coefficient (Cp), characteristic path length (Lp), global network efficiency (Eg), local network efficiency (Eloc), modularity index (Q), module number, and participation coefficient for the between‐group differences using two‐sample *t* tests (corrected *p* < .05). Additionally, we performed a correlation analysis between the Chinese language clear degree scale (CLCDS) scores and topological properties in children with NSCLP.

**Results:**

We detected significant between‐group differences in the areas under the curve (AUCs) of degree strength and betweenness centrality in language‐related brain regions. There were no significant between‐group differences in module number, participation coefficient, Cp, Lp, Eg, or Eloc. However, the Q (density: 0.05–0.30) and Q^AUC^ (*t* = 2.46, *p* = .02) showed significant between‐group differences. Additionally, there was no significant correlation between topological properties of statistical between‐group differences and CLCDS scores.

**Conclusions:**

Although nodal metric differences existed in the language‐related brain regions, the children with NSCLP after speech rehabilitation had similar global network properties, module numbers, and participation coefficient, but increased modularity. Our results suggested that children with NSCLP achieved speech rehabilitation through function specialization in the language‐related brain regions. The resting‐state topology pattern could be of substantive neurobiological importance and potential imaging biomarkers for speech rehabilitation.

## Graphical abstract

Our study found that the children with NSCLP after speech rehabilitation had similar global network properties, module numbers, and participation coefficient but increased modularity. Our results suggested that children with NSCLP achieved speech rehabilitation through function specialization in the language‐related brain regions. The resting‐state topology patterns could be of substantive neurobiological importance and potential imaging biomarkers for speech rehabilitation.

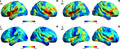

## 1 INTRODUCTION

Cleft lip and palate (CLP) is one of the most common craniofacial malformations in infants. It is estimated that the prevalence is 0.1% in live births (Centers for Disease and Prevention, 2006). CLP can be defined as two types, syndromic CLP and nonsyndromic CLP (NSCLP). Syndromic CLP is a portion of a well‐known syndrome, while NSCLP is not. Even following successful palatoplasty and pharyngoplasty, the percentage of compensatory articulation errors ranged from 5% to 50% (Taib et al., 2015). Speech therapy is the primary method to correct compensatory articulation errors caused by abnormal articulation placement, including the use of the normal velopharyngeal function, the formation of correct articulation patterns, and consolidation training (Chen, 2012). Speech therapy is usually combined with principles of motor learning by visual, auditory, and touch feedback assistance (Maas et al., 2008). However, the topological pattern after speech rehabilitation in children with NSCLP is poorly understood.

Several neuroimaging studies have identified both structural and functional brain abnormalities in patients with NSCLP after speech rehabilitation. An analysis of adults with NSCLP after articulation rehabilitation found the changes in the cortical thickness, gyrification, and fractal dimensions in the regions involved in language, auditory, pronunciation planning, and execution functions (Li et al., 2020). Another study of adult speech‐rehabilitated patients with CLP showed similar functional activation patterns as healthy controls, except for increased activation in the left hippocampus in a subvocalization task functional MRI study (Zhang et al., 2017). Our previous study found lower nodal shortest path length and higher nodal clustering coefficient of brain regions involved in higher‐order language and social cognition, and increased small‐world index of the whole brain in children with CLP after speech rehabilitation (Rao et al., 2020). Therefore, we performed a further study of the topological organization in the developing functional brain networks of children with CLP after speech rehabilitation.

Graph theory is a specific approach to investigate brain anatomical and functional networks. It has been widely applied in resting‐state functional MRI studies (Medaglia, 2017), such as primary progressive aphasia (Mandelli et al., 2016, 2018), adults who stutter (Ghaderi et al., 2018), and healthy people during infancy (Fan et al., 2011) and aging (Wu et al., 2012). Based on resting‐state BOLD signals characterizing physiological information of spontaneous neural activities in the brain (Biswal et al., 1995), we assess the local, modular, and global brain networks' characterizations using graph theory. For the local nodal properties, degree strength represents information on communication ability, and betweenness centrality describes the effect on the network's information flow. Modularity is widely accepted as one of the central organizing principles of the brain network (Bullmore & Sporns, 2009). It presents an optimal measure to balance the opposing requirements put on many changing systems: a great local specialization level, steady global integration, and the adaption of multiple or different selection criteria with time (Bullmore & Sporns, 2009; Kashtan & Alon, 2005). The detection and characterization of modular organization in the brain network can distinguish groups of anatomically and/or functionally related components that conduct specific biological functions. The participation coefficient measures intermodular connections describing a cost‐effective network integration (Bertolero et al., 2015). The global network properties, such as the local network efficiency (Eloc), global network efficiency (Eg), network clustering coefficient (Cp), and characteristic path length (Lp), represent the functional differentiation and integration of the whole‐brain network. Network efficiency (Eg and Eloc) is often disrupted by changes in path length (Bassett & Bullmore, 2009), and global cognitive function might depend on long‐distance connections (Lp) (Markov et al., 2013). Graph theory provides the ability to explore the local, modular, and global organization of the whole network, fundamentally different from other functional brain network analyses (Medaglia, 2017).

To our knowledge, few studies have explored the topological properties of functional brain networks in children with NSCLP. Therefore, the purpose of this study was to estimate the patterns in the topology of resting‐state networks between rehabilitated children with NSCLP and healthy controls using graph theory.

## 2 MATERIALS AND METHODS

### 2.1 Participants

The Beijing Children's Hospital and Beijing Stomatological Hospital ethical committee approved this study, and we obtained the informed consent of all children. Twenty‐eight children (age = 10.0 ± 2.3 years) with NSCLP and 28 age‐ and sex‐matched healthy controls were recruited from Beijing Children's Hospital and Beijing Stomatological Hospital. All children with CLP had already been evaluated by an experienced medical geneticist to exclude congenital syndromes. All children received a Chinese speech intelligibility test administered by three experienced speech pathologists. The children's inclusion criteria were as follows: (a) aged from 6–16 years old; (b) successful surgery of velopharyngeal insufficiency and speech therapy (Chinese language clear degree scale (CLCDS) scores > or =86); (c) normal hearing and vision (auditory brainstem response < 30 dB nHL); (d) average intelligence (the scores of Full‐Scale Intelligence Quotient (FSIQ) using the Chinese Wechsler Intelligence Scale for Children‐IV > 90); (e) Chinese as their mother tongue; and (f) right‐handed. The exclusion criteria for the patients were as follows: children with clinic diagnoses of (a) velopharyngeal anatomy or structure defect; (b) speech disorder (CLCDS scores < 86); (c) dysgnosia; (d) hearing and/or vision impairments; (e) congenital disorders; (f) developmental delays; and (g) other chronic health diseases.

### 2.2 Speech assessment

The CLCDS is a widely used method for the clear evaluation of speech in patients with CLP during speech therapy in China (Chen et al., 2002). One hundred Chinese words were selected that included the 21 consonants and all vowels by daily usage frequency to fill in a table, which contained all error‐prone consonants and vowels of patients with CLP. Eighty‐six correct phonetic words (or 86 points) were used as the cutoff point for the average level of clear Chinese speech, meeting daily oral communication (Wang et al., 1995). In children with NSCLP after speech rehabilitation, the CLCDS scores were 91.6 ± 4.0, reaching the average level of Chinese daily oral communication (see Table 1).

**TABLE 1:** Demographic and clinical characteristics

Sample members	Age		Boys/girls	CLCDS score
	Mean ± *SD*	Median	No.	Mean ± *SD*
NSCLP children	10.0 ± 2.3	9.6	21/7	91.6 ± 4.0
Healthy controls	10.4 ± 2.0	9.5	21/7	–

Abbreviations: CLCDS, Chinese language clear degree scale; HC, healthy controls; NSCLP, nonsyndromic cleft lip and palate.

### 2.3 Image acquisition

All MRI DICOM data were obtained with a 3.0T GE MRI system (with an eight‐channel phased‐array head coil) at the Department of Radiology (Beijing Children's Hospital). For each child, high‐resolution 3D T1‐weighted gradient‐echo anatomical and resting‐state functional MRI data were obtained. Using echo planner imaging (EPI) sequences, functional images were acquired with participants keeping their eyes closed while awake. The scan parameters of EPI were set as follows: repetition time = 2000 ms, echo time = 35 ms, flip angle = 90°, field of view = 240 mm × 240 mm, and in‐plane matrix = 64 × 64, which induces the spatial resolution = 3.75 mm × 3.75 mm, slice thickness = 3.0 mm, and slice gap = 1.0 mm. Each scanning session continued for 560 s, and each brain volume contained 38 axial slices. Structural images were acquired using a sagittal T1‐weighted three‐dimensional spoiled gradient sequence with the following parameters: 164 continuous sagittal slices, echo time = 3.516 ms, repetition time = 8.196 ms, slice thickness = 1 mm, flip angle = 13°, field of view = 256 mm × 256 mm, and matrix = 256 × 256.

### 2.4 Preprocessing

The obtained DICOM data were processed using the GRETNA toolbox software (http://www.nitrc.org/projects/gretna/) and Statistical Parametric Mapping 12 (SPM12, http://www.fil.ion.ucl.ac.uk/spm/) within the MATLAB environment. Briefly, the preprocessing of resting‐state functional MRI data involved the following steps: (a) the removal of the first 10 time point volumes for magnetization equilibrium; (b) slice timing correction because of the time offsets between slices; (c) rigid body correction of head motion; (d) spatial normalization for the Montreal Neurological Institute (MNI) space and resampling for 3‐mm isotropic resolution using a children's EPI template; (e) bandpass filtering (0.01 ~ 0.10 Hz); (f) linear detrending; and (g) linear regression for the white matter, global mean signal, cerebrospinal fluid signals, and six head motion parameters. No children were removed because of significant head motion (angular rotation ≥ 3° and/or displacement ≥ 3 mm) during the scan. Consequently, all children were included for further analysis.

### 2.5 Construction of the functional brain network

**FIGURE 1:**
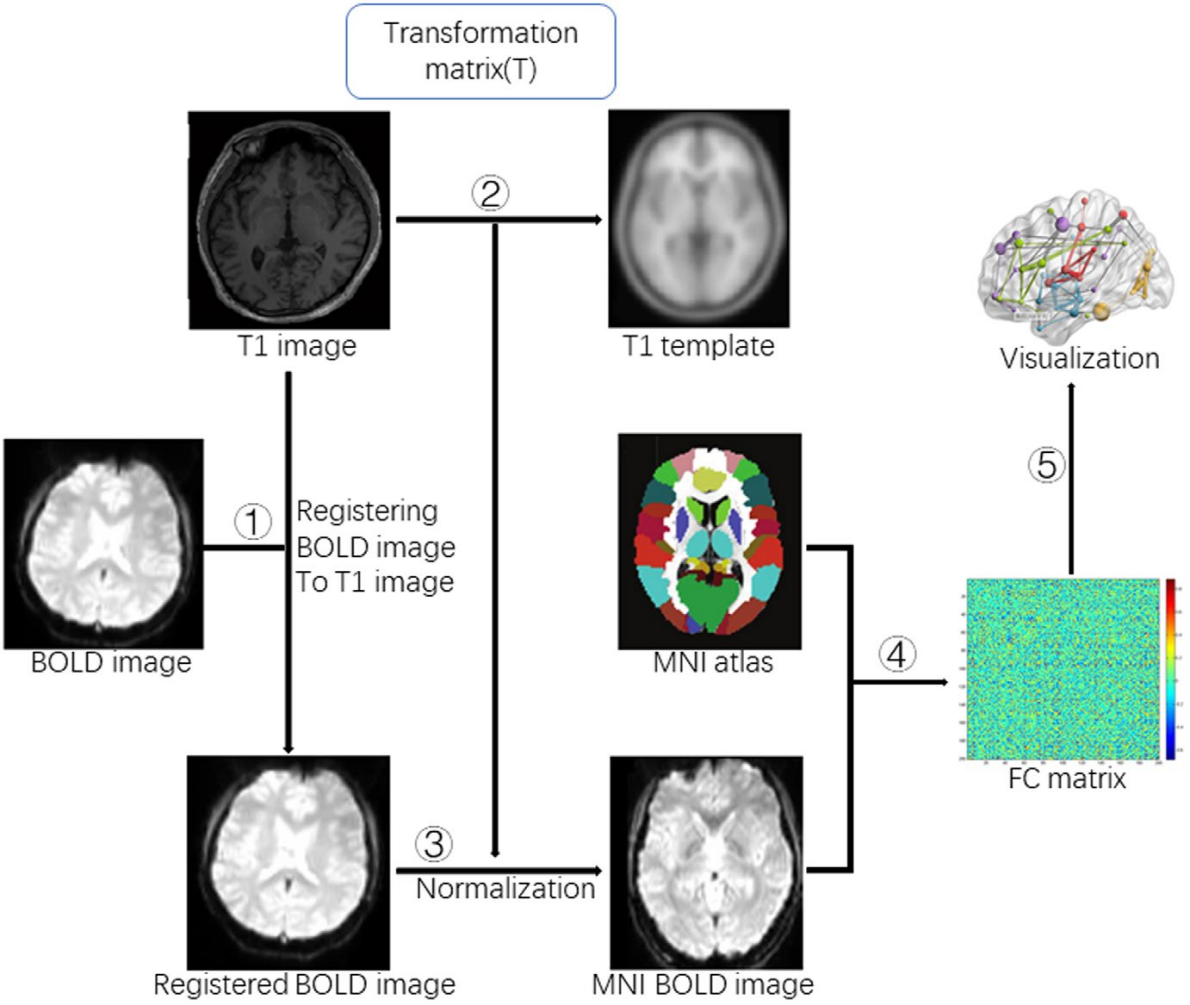
A flowchart of resting‐state network construction. ① Registering BOLD images to T1 weighted images, ② Transformation matrix (T), ③ Applying transformation matrix (T) for MNI BOLD images, ④ Extracting the mean time series of brain regions for FC matrix, ⑤ Visualization. MNI: Montreal Neurological Institute, FC: functional connectivity

Using the GRETNA toolbox software, the functional brain networks of all children were built. First, a 200 × 200 temporal Pearson correlation matrix was acquired by calculating the time‐series correlation coefficient between every pair of the 200 nodes (using the Harvard–Oxford Cortical Structural Atlas) for each participant (Craddock et al., 2012). During this step, the mean time series were computed by averaging all voxels' time series from each region. The values of the between‐region correlation coefficients were defined as the weights of the edges of the graph. Thus, a weighted function connection (FC) matrix (200 × 200) was obtained for each participant. Based on the FC matrix, the topological metrics of functional brain networks were examined. A series of threshold values were used to provide the same number of edges for each graph (see Figure 1). In this study, the range of threshold values was defined from 0.05 to 0.5 at the threshold interval of 0.01, and the metrics of the related graphs were evaluated at each threshold value (Zhang et al., 2011).

### 2.6 Network analysis

Graph theoretical analysis of children's weighted functional networks was assessed with routines in the GRETNA toolbox software. The topological network parameters were evaluated as follows: (a) nodal parameters: Local metrics included the degree strength and betweenness centrality of a node (Medaglia, 2017). The degree strength is equal to the sum of the edge weight between the node and its neighbors, which indicates its information communication capacity in the functional network. The betweenness centrality quantifies the extent to which a node participates in the shortest paths throughout the network, characterizing its effect on information flow between other nodes (b). Modular parameters: Modular structure metrics included the participation coefficient, module number, modularity index (Q), and the area under the curve (AUC) of modularity index (Q^AUC^) and participation coefficient (participation coefficient^AUC^), describing the best segmentation of the brain network into smaller modular functional communities (He & Evans, 2010). The Q is a measure used to quantificationally differentiate the number of intramodule connections in an actual network from that of a random network where connections are linked randomly (100 random networks applied in our study). The Q^AUC^ represents the Q values at all ranges of threshold values (Chen et al., 2011). The participation coefficient can also measure a node's significance in intermodular communication, describing the between‐module connection and communication, and participation coefficient^AUC^ represents the participation coefficient values at all ranges of threshold values (Tagliazucchi et al., 2013). A community structure could be considered a modular organization if *Q* ≥ 0.3 (Newman & Girvan, 2004), and connections were usually denser within modules than between different modules (Sheline et al., 2010) (c). Global parameters: Global network metrics included the global network efficiency (Eg), the local network efficiency (Eloc), the network clustering coefficient (Cp), and the characteristic path length (Lp). Eg is equal to the mean inverse shortest path length, describing the average minimal travel distance between nodes of the network, and Eloc is defined as the average of the global efficiencies of subgraphs comprising the nearby neighbors of a specific node (Latora & Marchiori, 2001). Cp is the mean clustering coefficient of the network's nodes, and Lp is defined as the mean shortest path through all pairs of nodes of the network (Watts & Strogatz, 1998). The weighted Eloc and Cp indicate network segregation of the brain, and Eg and Lp imply network integration of the brain.

### 2.7 Statistical analysis

The chi‐square test was applied for the between‐group difference in gender, and the two‐sample *t* test was used for between‐group difference in age. The whole density range's topological metrics and their areas under the curve (AUCs) were calculated. Age and sex were taken as covariates. The between‐group differences in topological parameters based on graph theory were performed for the statistical assessment using a series of two‐sample *t* tests with two‐tailed tests. Furthermore, the relationships between topological parameters and CLCDS scores in patients were computed using Pearson's correlation coefficient. In addition, the Bonferroni correction (corrected *p* <.05) was applied for multiple comparison corrections.

## 3 RESULTS

**FIGURE 2:**
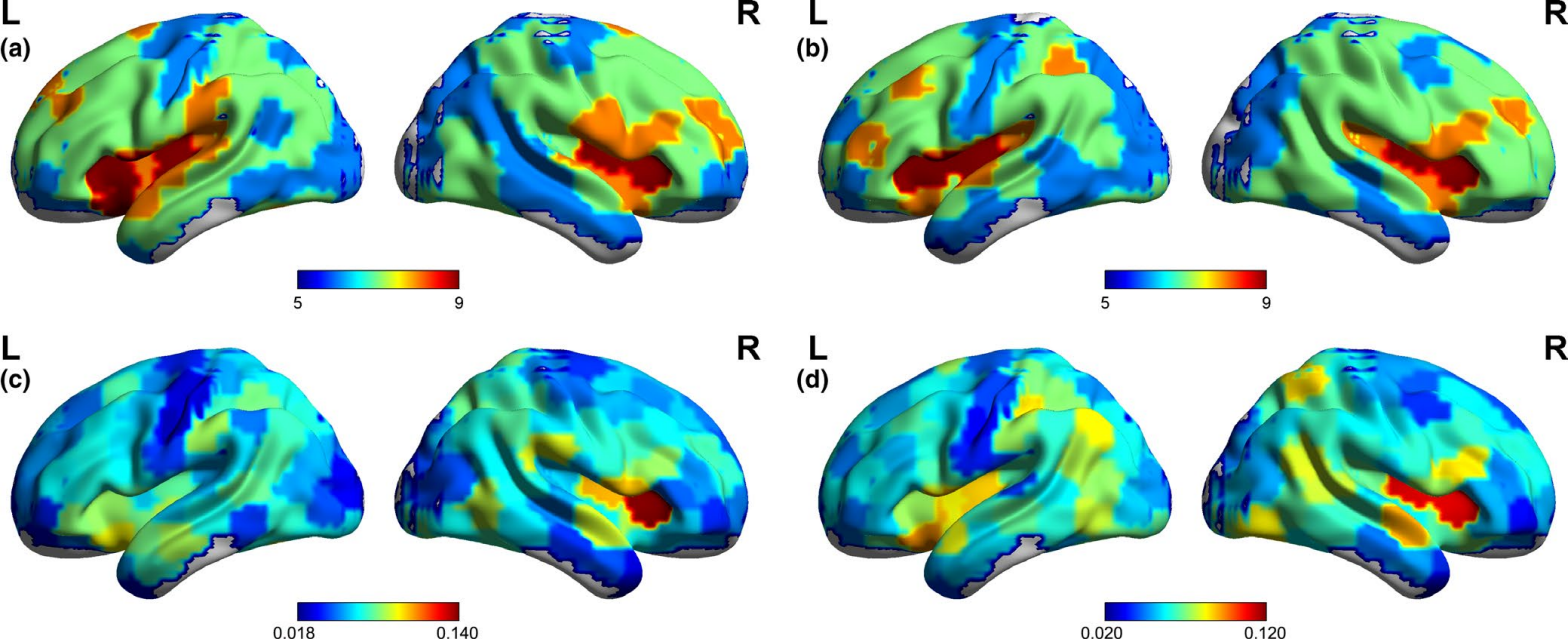
The projection of nodal values onto the cortical surface. a and b indicate the mean degree strength of the children with NSCLP and healthy controls, respectively. c and d indicate the mean betweenness centrality of the children with NSCLP and healthy controls, respectively. The color represents the nodal values

In this study, whole‐brain functional networks were constructed, and the local‐, modular‐, and global‐scale properties were evaluated for both children with NSCLP and healthy controls over the density range of 0.05–0.50 (step = 0.01). Interestingly, the local nodal metrics were mostly affected in the language‐related brain regions (Fujii et al., 2016). The mean nodal metric values of the two groups were projected onto the cortical surface (see Figure 2).

### 3.1 Demographic characteristics

The children's demographic characteristics are presented in Table 1. The children age of our study ranged from 6 to 16 years (10.0 ± 2.3), showing no significant between‐group differences (two‐sample *t* tests, *t* = −0.46, *p* = .96). The number of males was slightly higher than that of females in children with NSCLP and healthy controls, but the distribution showed no significant differences (chi‐square test, *χ* ^2^ = 0, *p* = 1) (see Table 1).

### 3.2 Between‐group differences in nodal degree strength and betweenness centrality

**FIGURE 3:**
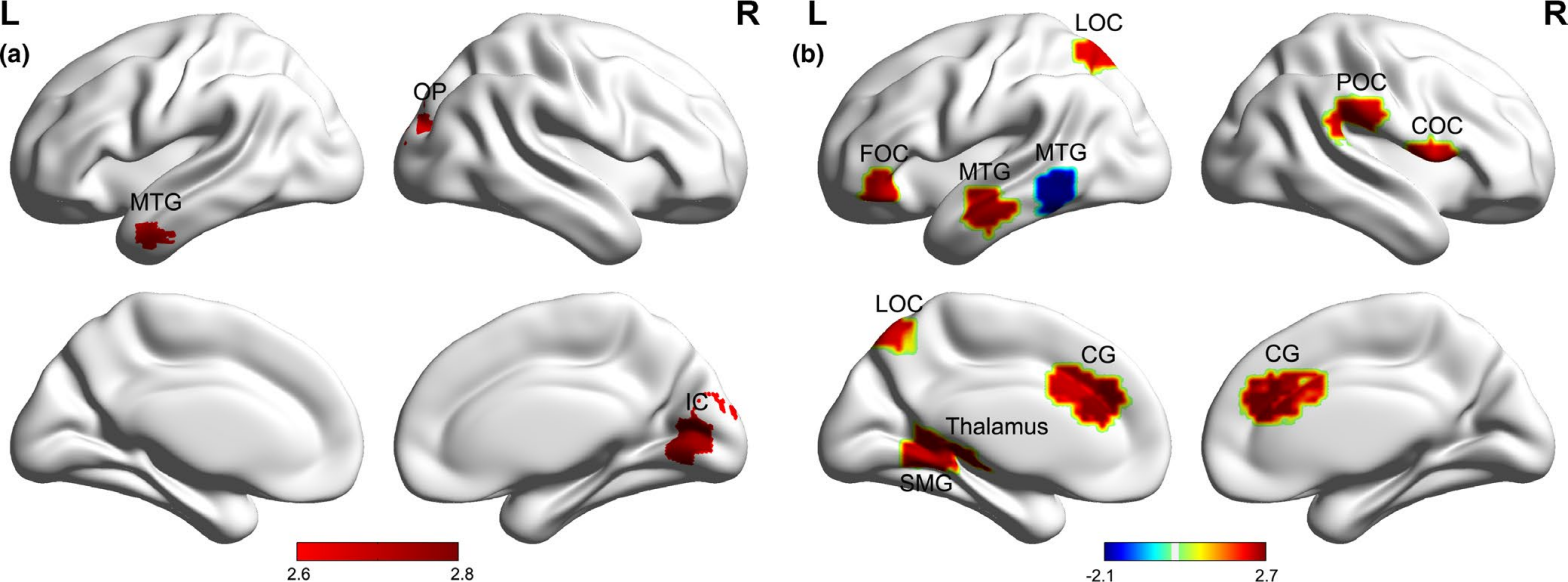
The distribution of the significant differences in the local nodal metrics. a, b: Between‐group differences in the AUC of the degree strength and betweenness centrality, respectively. Color represents the T value. Corrected for age and sex. Two‐sample *t* test, Bonferroni's correction, *p* < .05. AUC: area under the curve. MTG: middle temporal gyrus, OP: occipital pole, IC: intracalcarine cortex, LOC: lateral occipital cortex, FOC: frontal orbital cortex, COC: central opercular cortex, POC: parietal operculum cortex, SMG: supramarginal gyrus, CG: cingulate gyrus

Compared with healthy controls, children with NSCLP showed a higher degree strength^AUC^ in the left middle temporal gyrus of the Wernicke area, and in the right intracalcarine cortex and occipital pole of the primary visual center (see Table 2, Figure 3a).

**TABLE 2:** Significant differences in the local node metrics

Reg	Coordinates in RPI			Ds^AUC^	Bc^AUC^	Harvard–Oxford Cortical Structural Atlas
	*x*	*y*	*z*	*t* test value, *p*‐value		
37	−14	−30	0		2.591, .012	Left thalamus
40	1	35	23		2.723, .008	Left cingulate gyrus
55	0	18	32		2.411, .019	Left cingulate gyrus
72	−58	−13	−17		2.476, .016	Left middle temporal gyrus
74	−45	35	−9		2.324, .024	Left frontal orbital cortex
89	7	−72	5	2.760, 0.008		Right intracalcarine cortex
99	−59	−47	−9		−2.086, .041	Left middle temporal gyrus
101	−52	−2	−29	2.650, 0.010		Left middle temporal gyrus
119	55	7	5		2.233, .029	Right central opercular cortex
128	60	−29	25		2.264, .027	Right parietal operculum cortex
136	−15	−64	55		2.217, .034	Left lateral occipital cortex
142	17	−88	21	2.617, 0.011		Right occipital pole
179	−14	−51	−2		2.404, .019	Left supramarginal gyrus

Note

Reg is the code of the Harvard–Oxford Cortical Structural Atlas.

Abbreviations: AUC, area under the curve; Bc, betweenness centrality; Ds, degree strength

p^*^: *p*‐value with the Bonferroni correction.

For nodes with increased betweenness centrality^AUC^ in the children with NSCLP, compared with healthy controls, the central opercular cortex and parietal operculum cortex were located in the right cerebral hemisphere. The rest of the nodes with increased betweenness centrality^AUC^ were all in the left hemisphere and mostly in the dorsal stream of the neural basis of language (Fujii et al., 2016), such as the thalamus, anterior and posterior part of the cingulate gyrus, anterior part of the middle temporal gyrus, orbitofrontal cortex, lateral occipital cortex, and supramarginal gyrus. Only the left posterior part of the middle temporal gyrus showed decreased betweenness centrality (see Table 2, Figure 3b).

## 4 BETWEEN‐GROUP DIFFERENCES IN GLOBAL METRICS

**FIGURE 4:**
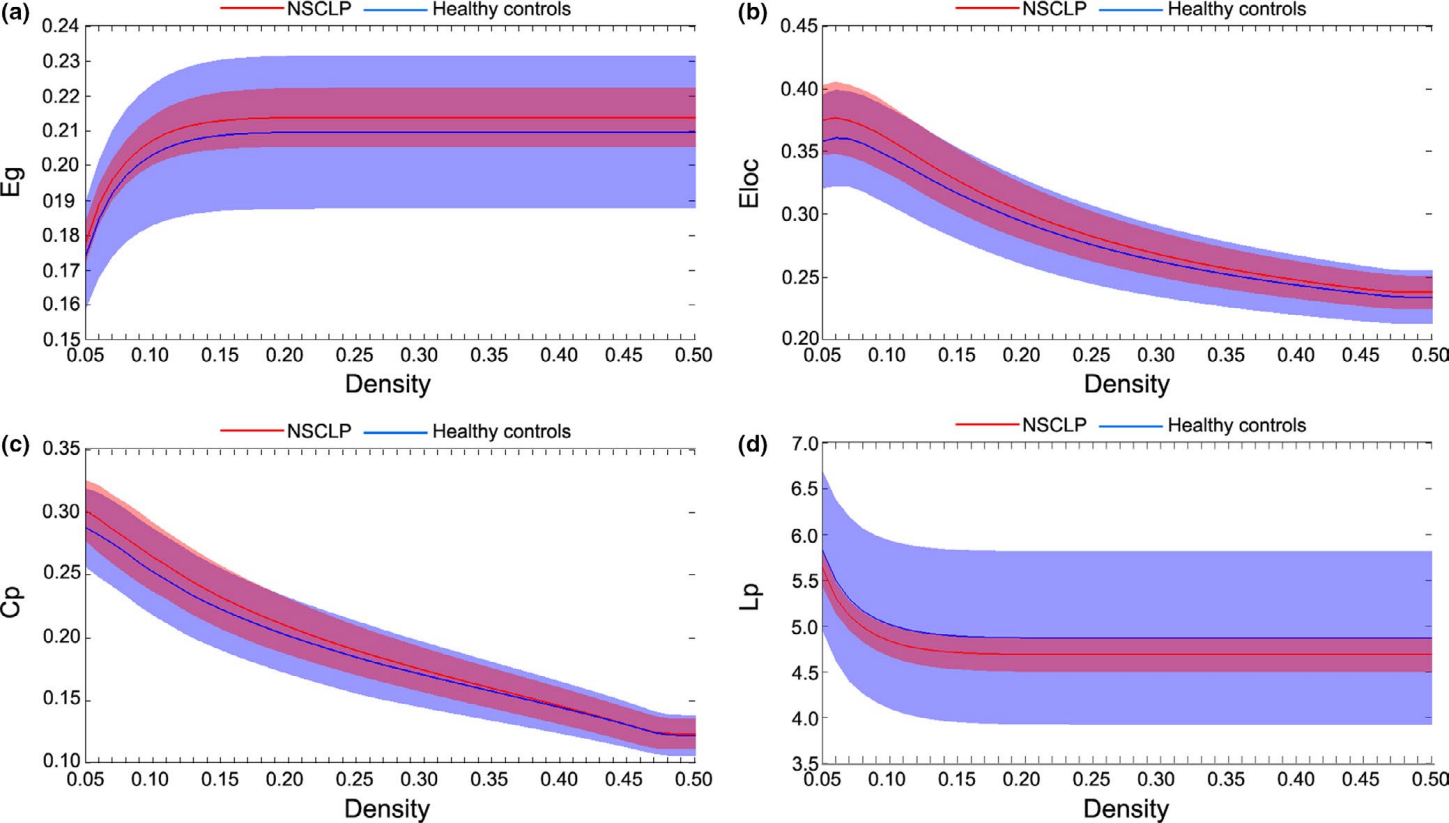
(a–d) Eg, Eloc, Cp, and Lp showed no significant differences. Shaded areas represent the standard deviation of the mean. Corrected for age and sex. Two‐sample two‐tailed *t* test. Bonferroni's correction, *p* < .05

There were no significant between‐group differences for C_p_^AUC^, L_p_^AUC^, Eg^AUC^, and E_loc_^AUC^ values of the network or Cp, Lp, Eg, and Eloc values of all threshold networks (see Table 3, Figure 4).

**TABLE 3:** Statistical analysis of modular and global metrics

		Lp	Cp	Eg	Eloc	Q^AUC^	Pc^AUC^
*T* test	*t*	0.02	2.26	0.21	1.28	2.46	1.49
	*p*	.88	.13	.65	.26	.02	.23
Correlation	*r*	0.04	−0.14	−0.05	−0.10	−0.24	−0.07
	*p*	.86	.48	.79	.60	.22	.72

Corrected for age and sex. *p* ^*^: *p*‐value with the Bonferroni correction.

Abbreviations: Cp, network clustering coefficient; Eg, network global efficiency; Eloc, network local efficiency; Lp, characteristic path length; Pc, participation coefficient; Q, modularity index.

### 4.1 Between‐group differences in modularity

**FIGURE 5:**
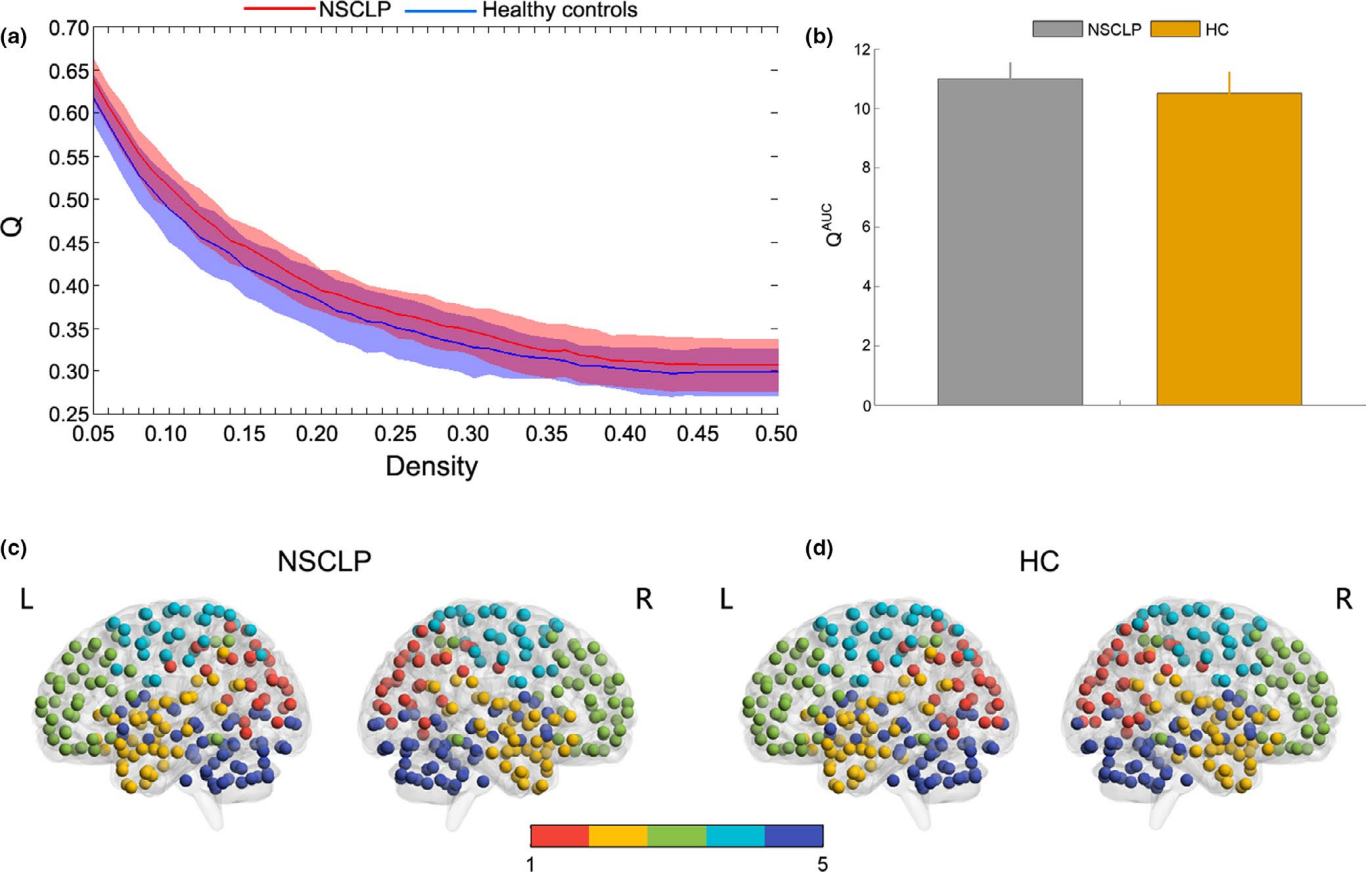
a: The Q showed significant between‐group differences at densities of 0.05–0.30. Shaded areas represent the standard deviation of the mean. b: The Q^AUC^ demonstrated significant between‐group differences. Columns and error bars represent the average Q^AUC^ values and their standard errors. The modularity structure of the group mean functional brain network in children with NSCLP (c) and HC (d) at density = 0.06. The children with NSCLP had five basic modules, similar to HCs. The color represents the number of modules. Corrected for age and sex. Two‐sample *t* test, Bonferroni's correction, *p* < .05. HC: healthy controls

At all threshold values, functional brain networks in children with NSCLP and healthy controls showed typical modular structure properties (*Q* > 0.3, see Figure 4a). Furthermore, compared with healthy controls, a two‐sample two‐tailed *t* test indicated that children with NSCLP exhibited a higher modularity index (density: 0.05–0.30; see Figure 5a), higher Q^AUC^ (*t* = 2.46, corrected *p* = .02; see Figure 5b), and no significant intergroup differences in module number and participation coefficient^AUC^ (*t* = 1.49, corrected *p* = .23). In addition, the brain networks of each functional connection matrix of children with NSCLP and healthy controls were decomposed into five basic modules (see Figure 5c,d).

### 4.2 Relationships between clinical characteristics and topological properties

Among the topological parameters of significant between‐group differences, there was no statistical correlation between CLCDS scores (see Table 3, Table S1).

## 5 DISCUSSION

To the best of our knowledge, this resting‐state functional MRI study was the first to explore brain networks' modularity in children with NSCLP. The major findings can be summarized as follows: (a) Nodal metric differences were mostly located in the language‐related brain regions; (b) brain networks of the two groups showed modularity and no significant between‐group differences in module number and participation coefficient^AUC^. However, the rehabilitated children with NSCLP showed higher Q and Q^AUC^ than healthy controls; and (c) global network metrics had no significant between‐group differences in Cp, Lp, Eg, or Eloc.

Among the nodes with increased degree strength, the middle temporal gyrus was involved in accessing the word/lexicon and its meaning (Saur et al., 2010; Schwartz et al., 2009). The right intracalcarine cortex and occipital pole were both located in the primary visual center. Our results implied that the communication capacity was strengthened in the middle temporal gyrus, right intracalcarine cortex, and occipital pole, which might help the circuit‐level calculation and total information transmission (Betzel et al., 2016). We inferred that the left middle temporal gyrus and the primary visual center might receive specific information from more brain areas for reestablishing correct articulation patterns and placements. Li et al. (2020) found increased gyrification located in the temporal lobe in the adults with NSCLP after speech rehabilitation compared with the healthy controls, consistent with our findings. In our study, speech therapy by visual feedback might improve the degree strength of the occipital cortex and middle temporal gyrus.

For nodes with increased betweenness centrality, most brain areas were located in the left hemisphere, which is the language‐dominant hemisphere. Our results showed that increased betweenness centrality was found in the left anterior part of the middle temporal gyrus, orbitofrontal cortex, lateral occipital cortex, and supramarginal gyrus. These brain areas were associated with phonological and semantic processing of language (Fujii et al., 2016). The posterior cingulate cortex provides “action” into the hippocampal memory system, and the anterior cingulate cortex (receiving from the orbitofrontal cortex) provides reward‐related input into the hippocampal memory system via the posterior cingulate (Rolls, 2019). The orbitofrontal cortex is involved in emotion and executive function (Rudebeck & Rich, 2018). In addition, the thalamus is the essential sensory conduction relay station and associates with perceptual, cognitive, and motor processes (Moustafa et al., 2017). The higher betweenness centrality for these brain areas suggested that the flows of language, emotion, and execution information increased (Kummer, 2011). We inferred that the children with NSCLP might be with the help of the language, motor, emotion, execution, and memory function for speech rehabilitation (measured by the CLCDS scores). Our previous study also found that the function of language‐related brain areas was higher (showing lower nodal shortest path length and higher nodal clustering coefficient) for children with NSCLP after speech rehabilitation compared with controls (Rao et al., 2020). Moreover, adult speech‐rehabilitated patients with CLP showed only increased activation in the left hippocampus in a subvocalization task functional MRI study (Zhang et al., 2017), which may be the pattern of speech rehabilitation in adults with NSCLP.

In addition, the only node with decreased betweenness centrality was located in the posterior part of the middle temporal gyrus (MTG). The posterior part of the MTG is a critical area in voice encoding, phonemic processes, and word selection processes during word expression (Glasser & Rilling, 2008). The betweenness centrality or information flows through the MTG in children with CLP were lower than those in healthy controls, which might indicate that after speech rehabilitation, word expression became clear, and the requirement diminished, so the flow of information was reduced through the posterior part of the MTG.

Modularity, one of the central organizing principles of complicated biological systems, has been widely used in recent years (Hartwell et al., 1999). Our findings confirmed the modular structure in resting‐state brain networks. In the two groups, five intrinsically cohesive modules were identified in the resting‐state functional networks, such as the default mode, sensorimotor, auditory, attention, visual, and salience networks, which were consistent with previous spontaneous brain activity studies (Mandelli et al., 2016, 2018). We detected modularity (*Q* > 0.3) in both groups and no significant between‐group differences in module number and participation coefficient^AUC^. The transmission and integration of information are the foundation of cognitive processing, which has been generally accepted. No significant between‐group differences in the module number and participation coefficient^AUC^ indicated that similar modules had been established in children with NSCLP compared with healthy controls. We know that integration among distant moduli was associated with rehabilitative cognitive functions (measured with CLCDS scores) (Bertolero et al., 2015). The similar module number and participation coefficient^AUC^ may indicate speech rehabilitation. However, the higher Q in rehabilitated children with NSCLP stated more intramodules for local information transfer, which suggested that functionally related components conduct specific biological functions with more specialization because of the adaption of habilitation. We presumed that the increased modularity index was linked with the higher degree strength and betweenness centrality induced by the repair surgery and speech therapy, which improved network adaption (Guye et al., 2010). Our previous study found the increased small‐world index in children with CLP after speech rehabilitation (Rao et al., 2020). We can infer that the higher modularity index caused the small‐world index to increase for the function specialization. Interestingly, our result was consistent with Duncan & Small's study, which stated that the increased modularity index of resting‐state brain networks was detected in patients after aphasia recovery (Duncan & Small, 2016).

Inspiringly, compared with healthy controls, rehabilitated children with NSCLP showed no significant differences in global network metrics of Cp, Lp, Eg, and Eloc. Our results indicated that rehabilitated children's global network metrics with NSCLP exhibited the average level from abnormal topological properties. Clinically, the CLCDS scores (no less than 86) of children with NSCLP meant speech rehabilitation. This implied that the functional integration and differentiation of the whole‐brain networks in rehabilitated children with NSCLP might be consistent with those in healthy controls individually. A subvocalization task fMRI study showed similar functional activation patterns between speech‐rehabilitated adults with CLP and healthy controls (Zhang et al., 2017). Another study demonstrated the global topological parameters partially recovered to levels similar to those of healthy controls in patients with aphasia after speech treatment (Baliki et al., 2018). The two studies also supported our results. The network global metrics and CLCDS scores confirmed our hypothesis that the rehabilitated children with NSCLP would be characterized by a similar functional topological organization to that of healthy controls.

## 6 LIMITATIONS

This study still has its limitations. First, the number of children in both groups was relatively small. Second, more work focused on the investigation of the differences in the topological organization of the functional networks in children with NSCLP before speech rehabilitation should be done in the future. Third, although the exclusion criteria of our study cannot exclude all inherent and acquired factors of impaired brain development, speech therapy may have primary effects on the brain for speech rehabilitation. Fourth, a longitudinal study of the alterations identified in language‐related areas and networks in speech‐rehabilitated children with NSCLP could be conducted. Fifth, the CLCDS scores in healthy controls should be estimated for the between‐group difference in the future, contributing a lot to our results. Sixth, it is important to note that given the study design (i.e., no pretherapy measures and no measures of participants who did not achieve adequate speech following therapy), a causal relationship between speech therapy and topology patterns cannot yet be inferred. However, the findings of increased network modularity for participants with NSCLP following speech therapy support the need for further research in this area.

## 7 CONCLUSION

There were no significant differences in global network metrics for children with NSCLP after speech therapy. However, significant differences existed in local nodal metrics for the language‐related brain regions. In addition, the NSCLP group had increased network modularity (Q and Q^AUC^) compared with the healthy controls. The similar global network metrics and increased network modularity provided profound insights into the neurobiological understandings of speech‐rehabilitated children with NSCLP and could be potential imaging biomarkers for the estimation of speech rehabilitation.

## CONFLICT OF INTEREST

The authors declare that they have no conflicts of interest.

## AUTHOR CONTRIBUTIONS

Bo Rao designed methodology, provided software, and wrote the original draft. Hua Cheng wrote, reviewed, and edited the manuscript; administered the project; validated the data; and acquired funding. Wenjing Zhang involved in formal analysis and curated the data. Renji Chen investigated the study and provided resources. Yun Peng supervised the study and acquired funding.

### PEER REVIEW

The peer review history for this article is available at https://publons.com/publon/10.1002/brb3.2094.

## Supporting information

Table S1

## Data Availability

In our study, we used and analyzed the datasets, which is available from the corresponding author for a reasonable request.
