# Supplementary material for: Increased modularity of the resting‐state network in children with nonsyndromic cleft lip and palate after speech rehabilitation
Source: Brain Behav. 2021 Aug 3;11(8):e02094. doi: 10.1002/brb3.2094 (PMC8413807; doi:10.1002/brb3.2094)
Supplement: Supplementary file 1 — Table S1 [file BRB3-11-e02094-s001.docx]

Table S1 Correlation analysis of nodal metrics with the CLCDS scores.

| Reg |  | Coordinates in RPI | | |  | Ds^AUC^ | Bc^AUC^ |  | Harvard-Oxford Cortical Structural Atlas |
| --- | --- | --- | --- | --- | --- | --- | --- | --- | --- |
|  |  | x | y | z |  | *r* value, *p*^*^ value | |  |  |
| 37 |  | -14 | -30 | 0 |  |  | 0.046, 0.816 |  | Left Thalamus |
| 40 |  | 1 | 35 | 23 |  |  | -0.155, 0.432 |  | Left Cingulate Gyrus |
| 55 |  | 0 | 18 | 32 |  |  | 0.201, 0.306 |  | Left Cingulate Gyrus |
| 72 |  | -58 | -13 | -17 |  |  | -0.142, 0.471 |  | Left Middle Temporal Gyrus |
| 74 |  | -45 | 35 | -9 |  |  | 0.115, 0.559 |  | Left Frontal Orbital Cortex |
| 89 |  | 7 | -72 | 5 |  | 0.367, 0.055 |  |  | Right Intracalcarine Cortex |
| 99 |  | -59 | -47 | -9 |  |  | -0.020, 0.919 |  | Left Middle Temporal Gyrus |
| 101 |  | -52 | -2 | -29 |  | -0.069, 0.728 |  |  | Left Middle Temporal Gyrus |
| 119 |  | 55 | 7 | 5 |  |  | 0.318, 0.067 |  | Right Central Opercular Cortex |
| 128 |  | 60 | -29 | 25 |  |  | -0.067, 0.734 |  | Right Parietal Operculum Cortex |
| 136 |  | -15 | -64 | 55 |  |  | 0.362, 0.059 |  | Left Lateral Occipital Cortex |
| 142 |  | 17 | -88 | 21 |  | 0.207, 0.291 |  |  | Right Occipital Pole |
| 179 |  | -14 | -51 | -2 |  |  | -0.053, 0.787 |  | Left Supramarginal Gyrus |

Ds: degree strength, Bc: Between centrality. CLCDS: Chinese language clear degree scale. Corrected for age and sex. *p*^*^: p-value with the Bonferroni correction.
